# Factors Associated With Non-suicidal Self-Injury in Chinese Adolescents: A Meta-Analysis

**DOI:** 10.3389/fpsyt.2021.747031

**Published:** 2021-11-30

**Authors:** Yang-yang Fan, Jing Liu, Yan-yan Zeng, Rachel Conrad, Yi-lang Tang

**Affiliations:** ^1^Department of Public Administration, School of International and Public Affairs, Shanghai Jiao Tong University, Shanghai, China; ^2^The National Clinical Research Center for Mental Disorders, Beijing Key Laboratory of Mental Disorders, Beijing Anding Hospital, Capital Medical University, Beijing, China; ^3^Advanced Innovation Center for Human Brain Protection, Capital Medical University, Beijing, China; ^4^Department of Clinical Psychology, Shenzhen Kangning Hospital, Shenzhen, China; ^5^Department of Psychiatry, Harvard Medical School, Boston, MA, United States; ^6^Department of Psychiatry, Brigham and Women's Hospital, Boston, MA, United States; ^7^Department of Psychiatry and Behavioral Sciences, Emory University School of Medicine, Atlanta, GA, United States; ^8^Mental Health Service Line, Atlanta VA Medical Center, Decatur, GA, United States

**Keywords:** non-suicidal self-injury, adolescents, meta-analysis, China, associated factors

## Abstract

Non-suicidal self-injury (NSSI) in adolescents is an increasing public health problem in China. We conducted a meta-analysis of studies on NSSI in Chinese adolescents (between 10 and 19 years) to examine factors associated with NSSI. Twenty-five papers including 30 separate studies with 186,447 participants were included for analysis. The results from a random-effects model showed a weak, but significant overall prediction of NSSI (OR = 1.734). There were significant associations between the following seven factors and NSSI (ranking by the effect sizes, in descending order): adverse life events (OR = 2.284), negative coping style (OR = 2.040), problematic internet use (OR = 2.023), sleep disturbance (OR = 1.734), traumatic experiences (OR = 1.728), problematic parent-child relationship (OR = 1.585), mental health problems (OR = 1.578). Additionally, NSSI sample type moderated these effects. These findings highlight factors significantly associated with NSSI in Chinese adolescents. Parent-child relationship and mental health of the only children and left-behind children in China deserve more attention.

**Systematic Review Registration:** https://www.crd.york.ac.uk/prospero/display_record.php?ID=CRD42019123508.

## Introduction

Non-suicidal self-injury (NSSI) is common among adolescents and it poses a major public health concern (1–3). NSSI is generally defined as self-directed, intentional behavior that causes physical injuries but without suicidal intent. The common types of NSSI include cutting, scratching, hitting, banging, and burning oneself (4). NSSI often causes concerns and distress for parents, teachers, or mental health professionals working with the affected adolescents. Some data suggest that prevalence of NSSI is increasing in China in recent years (5) and a recent meta-analysis (conducted in 2018) found that the prevalence of NSSI among middle-school students in China was 22.37% (95% CI: 18.84–25.70%) (4).

Current models based on identified risk factors predict only a small proportion of NSSI, and there are no validated clinical risk assessments with high predictive value for future NSSI (6).

NSSI is a significant public health concern due to its association with many poor outcomes. NSSI is a significant predictor of future suicide attempts in adolescents (7, 8). One study found that rate of suicide for patients within 1 year of NSSI was 66 times higher than the rate of suicide in the general population (9). NSSI is associated with a wide range of psychiatric disorders including post-traumatic stress disorder, eating disorders (10), substance use disorders (11) and borderline personality disorder in adults (12).

According to the definition of adolescence by the World Health Organization (roughly between the ages of 10 and 19 years), this period begins with the onset of physiologically normal puberty, and ends when an adult identity and behavior are accepted (13). Adolescence is a period with vulnerability to NSSI, possibly because of adolescents' impulsiveness and emotional reactivity associated with brain development (14). The prevalence of NSSI among adolescents in the general population ranges from 17 to 31% (15, 16). NSSI is more prevalent in adolescents seeking psychiatric treatment than in the general population, and up to 60% of adolescents admitted to psychiatric units report having engaged in at least one NNSI episode during their lifetime, and 50% report recurrent NSSI (14).

Engagement in NSSI can serve a range of functions for the individual, including emotion-regulation, self-punishment, communication of distress, punishing others, or influencing others (17). NSSI is often multifactorial and is associated with cultural factors, interpersonal stressors, neurobiological background, emotional dysregulation and adverse childhood experiences (1).

Understanding factors associated with NSSI is critical to developing both clinical risk assessments and therapeutic interventions. A few reports have examined factors associated with NSSI in Western culture, including a meta-analysis (18, 19). However, the few existing meta-analyses do not include large studies from China (18). After more than three decades of implementation of the one-child policy, it is estimated that there are at least one hundred million of the population are only children in China (20). The policy was replaced by a universal two-child policy in 2015 (21), and this may lead to changes in population composition. In addition, left-behind children are a special group of children living in rural China whose parents have migrated to cities in search of work (22). Parent-child separation has been associated with a range of negative long-term physical and mental health outcomes, although many studies of children who are separated from parents involve the compounding traumatic exposure of war (23–25). The issues related to left-behind children have aroused widespread concern in Chinese society currently (26). Factors related to NSSI may be specific to culture or population, and it is important to explore factors associated with NSSI in adolescents in China.

This meta-analysis reviews existing publications pertaining to NSSI among adolescents in China to examine the factors associated with NSSI in this population.

## Materials and Methods

### Search Strategy

We searched the following databases: PubMed, Scopus, Medline, PsycINFO, Embase, Cochrane Library, Chinese National Knowledge Infrastructure (CNKI), and *Wanfang* for English and Chinese-language publications between January 1, 2000 to June 30, 2020. The search terms were: “self-injury/self-harm/self-mutilation/self-abuse/deliberate self-harm (DSH)/ non-suicidal self-injury/NSSI” AND “adolescent/teen/students/youth” AND “China/Chinese.” Additional eligible studies were added from the search of reference lists included in relevant literature reviews, systematic reviews, and meta-analyses of NSSI.

### Selection Criteria

Cross-sectional or longitudinal studies were included. The including criteria were: (1) focused on Chinese adolescents (or reported age between 10 and 19 years); (2) reported a definition of NSSI and the measure of the relative risk for NSSI over the previous 12 months (including less than 12 months) and (hazard ratios, relative risks, and odds ratios [ORs]) associated with a predictive factor and a corresponding measure of variability; (3) published in Chinese or English languages; (4) for longitudinal studies, the including participants need to be matched between the first time and the follow-up assessment; (5) reported NSSI behavior rather than suicidal attempts; (6) peer-reviewed journal articles only. Review articles, commentaries, case reports, editorials, animal studies, and meta-analysis were excluded.

### Data Extraction and Quality Assessment

In order to assess the quality of the publications, some essential characteristics were extracted by two investigators (Y-YF and Y-YZ) independently using an excel form: The first author's name, year of publication, study design, location of the study, diagnostic method, population size, the sample size of NSSI, sample type, the average age of subjects, covariates in multivariate analysis, multivariable-adjusted estimates and the 95% CIs (see Supplementary Table 1). Both authors are familiar with the procedures of meta-analysis and the concept of NSSI, and the intercoder reliability is 92.47% (Cohen's Kappa Coefficient). The raw data were used to transform into effect size if studies did not report the risk estimates or other estimates (OR, RR, or HR). For quality assessment, we used the Agency for Healthcare Research and Quality Checklist (27) to assess the cross-sectional studies and The Newcastle Ottawa Scale (NOS) (28) was used to assess longitudinal studies. For inconsistent coding and quality assessment, the article was checked by the two investigators and discussed with the corresponding author (JL). All coding divergences were resolved and reached a consistent conclusion through these processes (see Supplementary Tables 2, 3).

### The Strategy of Meta-Analyses

In this meta-analysis, Stata 16 and Comprehensive Meta-Analysis 3.0 were used for data analysis. Cochran's Q test and I^2^ statistics were applied to test heterogeneity among those studies. If the significant Q-value was greater than the degree of freedom and the I^2^ was bigger than 75%, that means the heterogeneity is significant. The random-effects model was conducted if the heterogeneity was significant (29). By using subgroup analysis, we compared the differences among those factors to explain heterogeneity. Meta-regression analysis was conducted based on Stata16 for testing the moderator effect. We use Egger's test of the intercept to evaluate publication bias, and we also used the funnel plots to analyze the publication bias.

## Results

### Search Results and Characteristic of Studies

**Figure 1:**
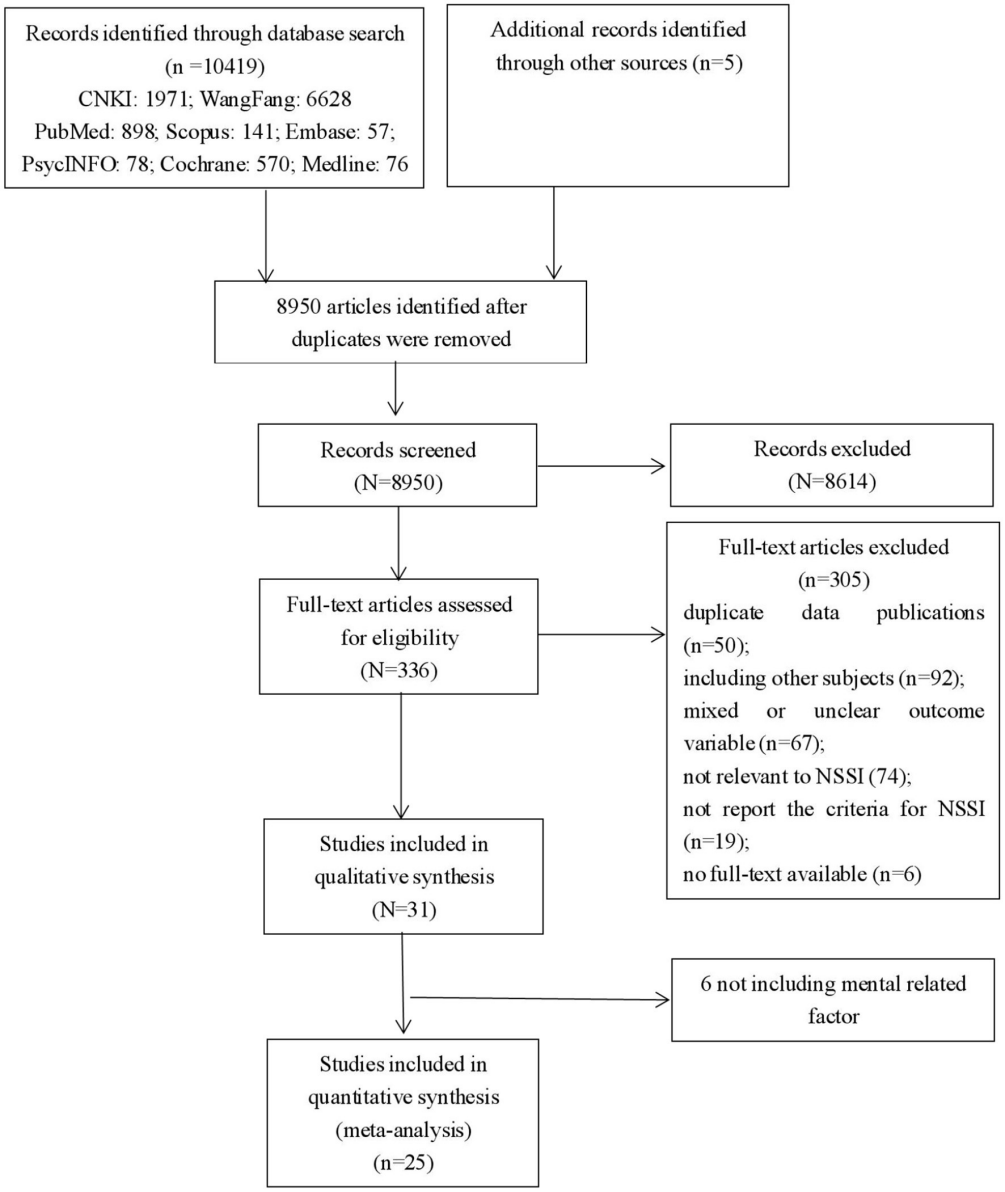
PRISMA flow chart for meta-analysis of publication on non-suicidal self-injury in adolescents.

As shown in Figure 1, a total of 10,424 articles were included at the initial stage, and 8,950 articles were identified after duplicates were removed. After the initial screening involving reviewing the titles and the abstracts, 336 papers were progressed to full-text evaluations. After the assessment of eligibility, 305 papers were excluded for the following reasons: duplicate data publications (*n* = 50), including other subjects (*n* = 92), mixed or unclear outcome variable (*n* = 67), was not relevant to NSSI (*n* = 74), did not report the criteria for NSSI (*n* = 19), no full-text available (*n* = 6). Eventually, 31 studies were included in qualitative synthesis, and as we only focused on the psychological risk factors, 25 full-text articles including 30 separate studies (94 prediction cases) with 186,447 subjects were included in this meta-analysis (Figure 1; Supplementary Table 1). Quality assessment of included cross-sectional and longitudinal studies were shown in Supplementary Tables 2, 3 (Quality assessment score should be greater than 4 points). Among those 30 separate studies, the average sample size was 6,929 (SD = 6,401) with the minimum (650) and maximum (25,378) samples. The total participants number of those 30 studies were 207,868 with 56,624 samples reported NSSI (27.24%). We extracted more details about including studies and presented them in the Supplementary Table 1.

### Factors Associated With NSSI

#### Traumatic Experience

**Figure 2:**
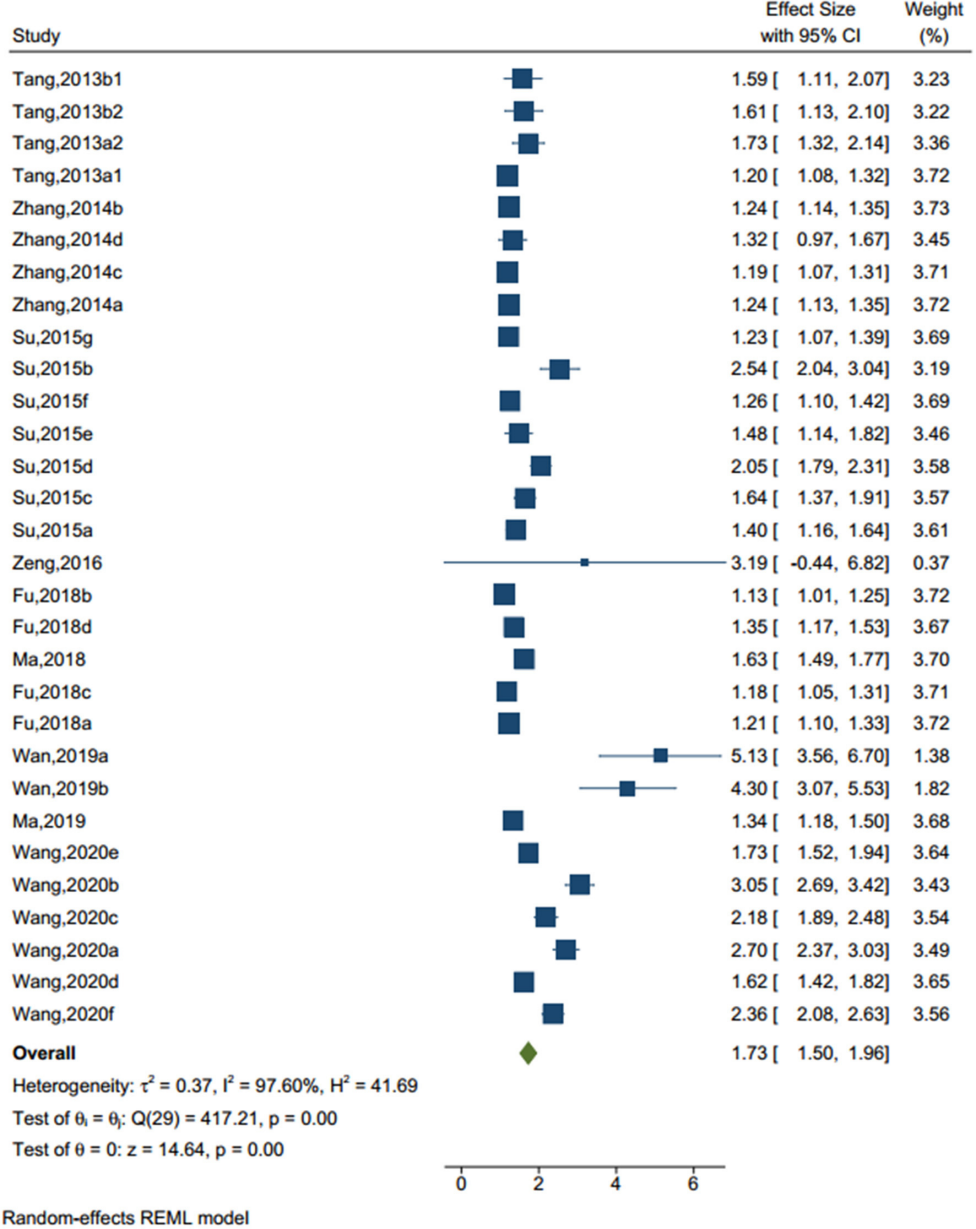
Forest plot of studies including traumatic experience factor.

There were 30 studies (sample size=73,423) identified a traumatic experience (see Figure 2), such as adverse childhood experiences, general trauma, emotional abuse, sexual trauma, emotional neglect, indirect aggression, verbal aggression, verbal abuse, and physical abuse. The random-effect model showed that, compared to those without traumatic experience, the odd ratio (OR) to have NSSI in adolescents who had traumatic experience was 1.728 (95% CI = 1.496–1.959, *I*^2^ = 97.60%, *p* < 0.001).

#### Problematic Parent-Child Relationship

**Figure 3:**
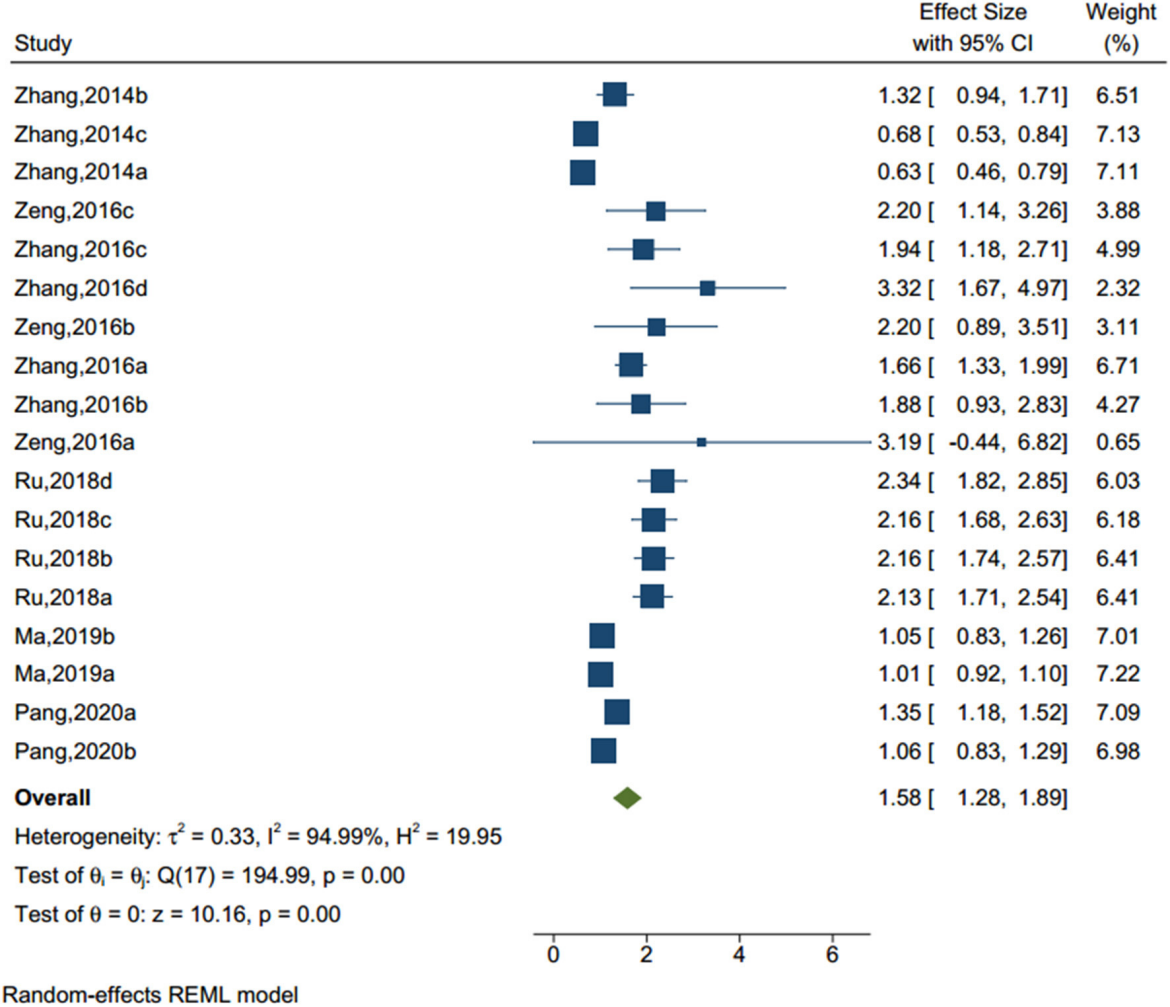
Forest plot of studies including problematic parent-child relationship factor.

Eighteen studies (sample size = 456,540) identified a problematic parent-child relationship (see Figure 3), including neglect, harsh punishment, and intrusiveness from parents. The results of the homogeneity test showed that there was significant heterogeneity (Q-value = 194.99, I^2^ = 94.99%, *p* < 0.001). The random-effect model showed that adolescents with unhealthy parent-child relationship had an OR of 1.585 to have NSSI compared to those without (95% CI = 1.279–1.899, *p* < 0.001).

#### Adverse Life Events

**Figure 4:**
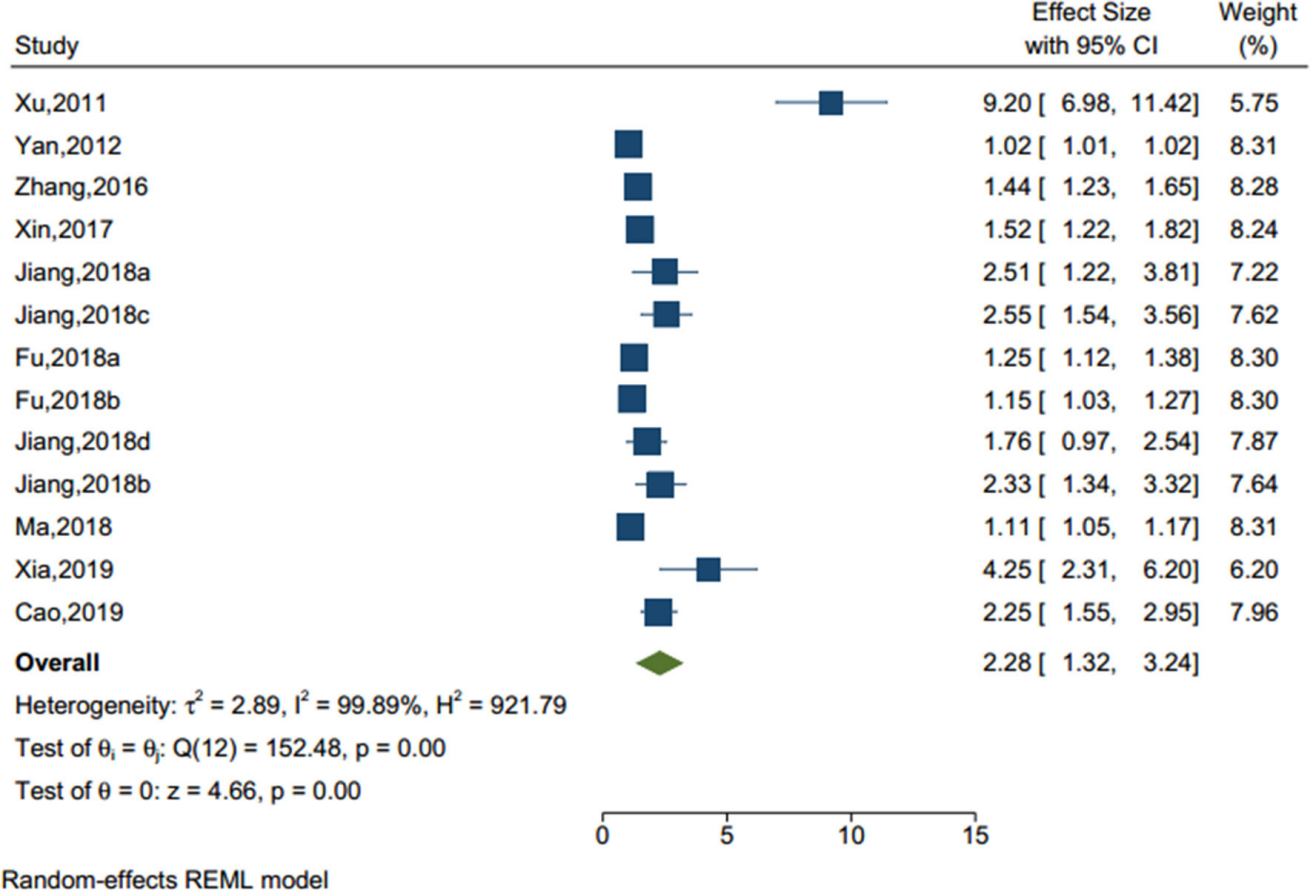
Forest plot of studies including adverse life events factor.

Thirteen studies (sample size = 42,574) identified an adverse life events (see Figure 4), including left-behind experiences (children under 18 who are left behind in rural areas when one or both parents migrate to an urban area for work for at least 6 months) (30), bullying, stressful life event, and self-reported academic pressure. The result showed that there was a significant effect of adverse life events on NSSI (OR = 2.284, 95% CI = 1.324–3.245, I^2^ = 99.89%, *p* < 0.001). Of note, among those seven factors, adverse life events were the most significant factors associated with NSSI.

#### Sleep Disturbance

**Figure 5:**
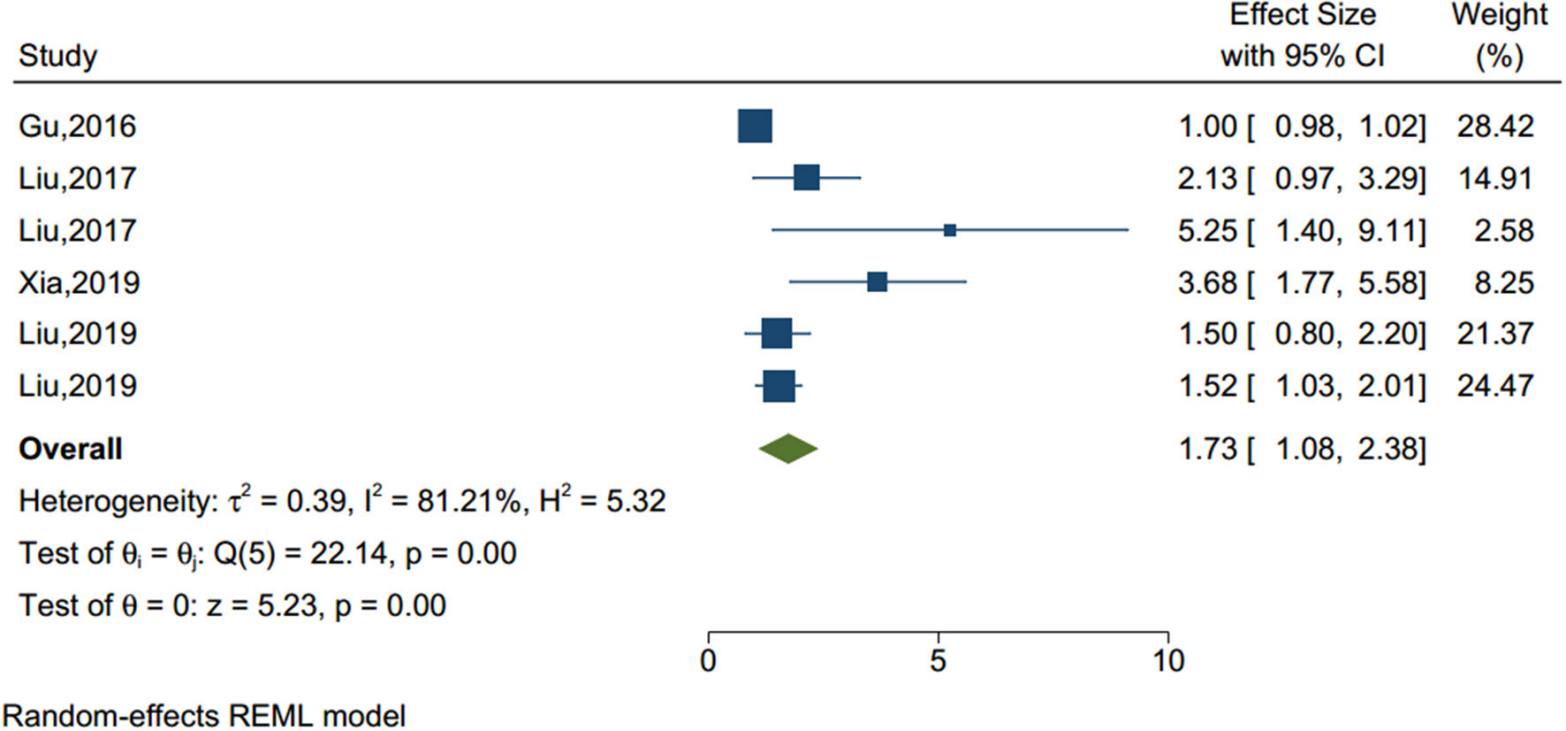
Forest plot of studies including sleep disturbance factor.

Poor sleep quality and frequent nightmares were identified as an important factor of NSSI in previous studies (see Figure 5). Based on the six studies (sample size = 15,611), our analysis showed that sleep disturbance was a significant factor associated with NSSI (OR = 1.734, 95% CI = 1.084–2.383, *I*^2^ = 81.21%, *p* < 0.001).

#### Problematic Internet Use

**Figure 6:**
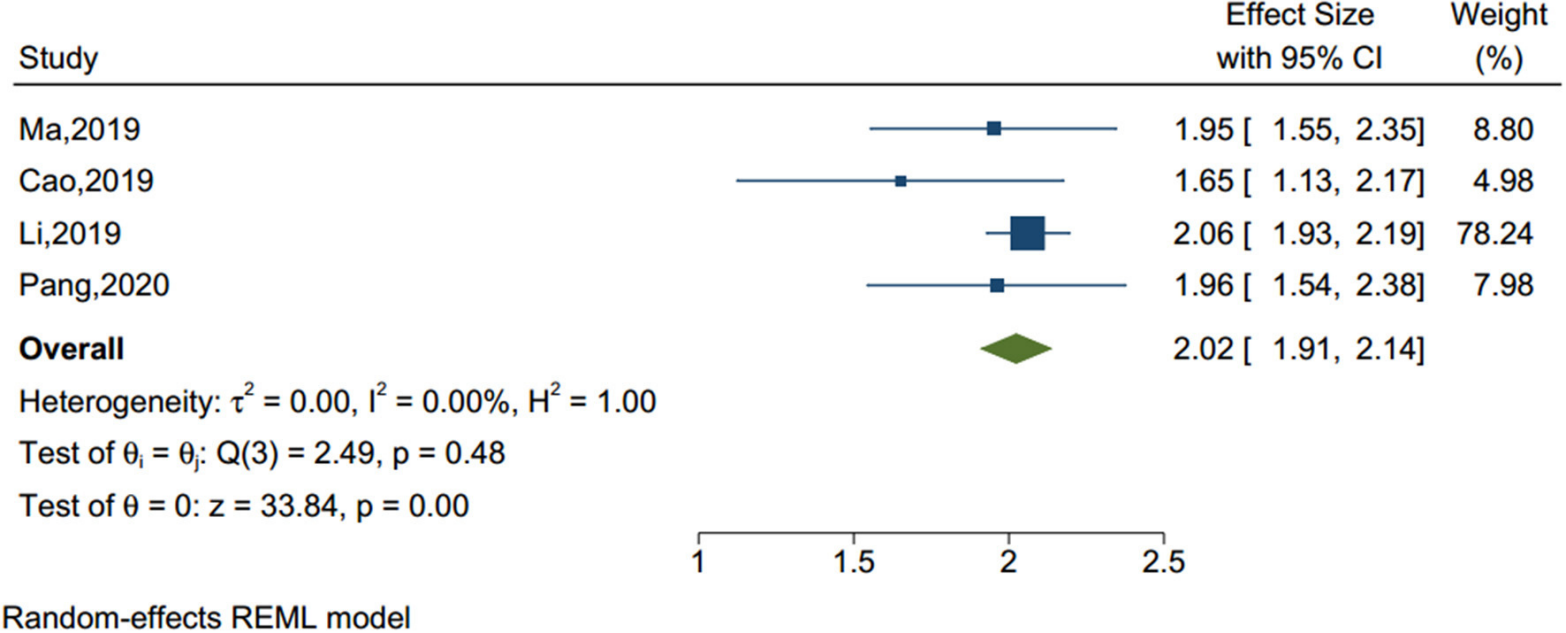
Forest plot of studies including problematic internet use factor.

Four studies (sample size = 55,092) identified internet addiction (see Figure 6), including problematic mobile phone and internet use. As shown in Table 1, the Q-value was lower than the degree of freedom [Q-value = 2.49, df(Q) = 3, *p* = 0.477] and the I^2^ was <75%, therefore the fix effect model was applied. The analysis showed that adolescents with internet addiction had more than two times the odds of NSSI than those without (OR = 2.023, 95% CI = 1.906–2.141, *p* < 0.001).

**Table 1:** The results of homogeneity test.

**Factors**	**Prediction cases**	***n***	**Heterogeneity**				
			**Q-value**	**df(Q)**	***p* value**	***I* ^**2**^**	**Tau^**2**^**
Traumatic experience	30	73,423	417.21	29	0.000	97.60	0.371
Problematic parent-child relationship	18	45,654	194.99	17	0.000	94.99	0.335
Adverse life events	13	42,574	152.48	12	0.000	99.89	2.888
Sleep disturbance	6	15,611	22.14	5	0.001	81.21	0.386
Problematic internet use	4	55,092	2.49	3	0.477	0.00	0.000
Mental health problems	17	93,120	796.26	16	0.000	99.98	0.644
Negative coping style	6	21,584	100.15	5	0.000	97.77	0.820
Overall	94	186,447	2,445.91	93	0.000	99.93	0.484

#### Mental Health Problems

**Figure 7:**
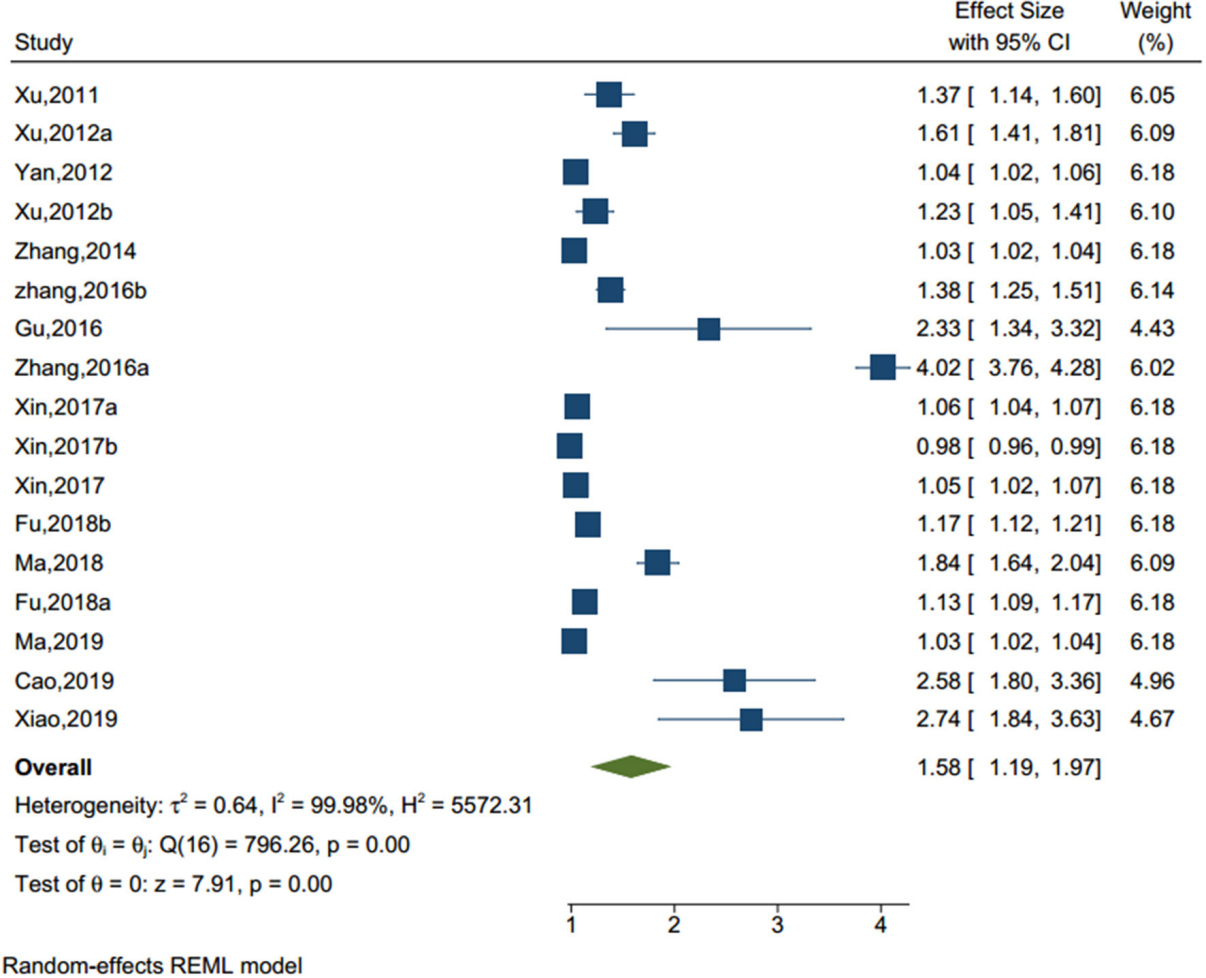
Forest plot of studies including mental health problems factor.

With significant heterogeneity (I^2^ = 99.98%, *p* < 0.001) among 16 studies (sample size = 89,163) (see Figure 7), adolescents with mental health issues had more than 1.5 times the risk of NSSI (OR = 1.594, 95% CI = 1.177–2.011) than those without mental health issues. The common mental health issues examined included loneliness, subclinical mental health issues, emotional problems, anxiety, and fear.

#### Negative Coping Style

**Figure 8:**
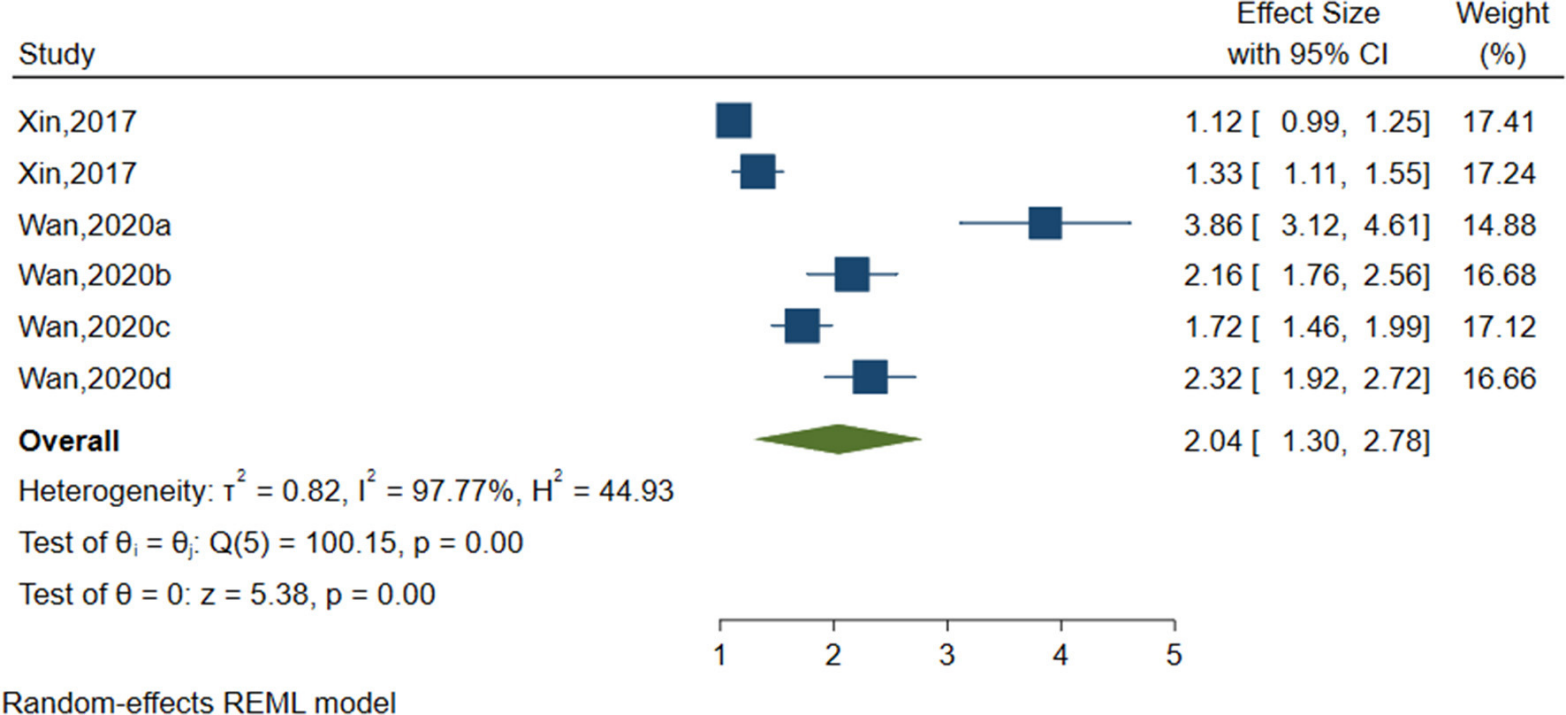
Forest plot of studies including negative coping style factor.

Six studies (sample size = 21,584) identified negative coping styles (see Figure 8) including fighting, physical inactivity and negative coping style in general. As shown in Table 2, negative coping styles were significantly associated with NSSI (OR = 2.040, 95%CI = 1.297–2.783, *I*^2^ = 97.77%, *p* < 0.001).

**Table 2:** Result of all factor analysis of included studies in the meta-analysis.

**Factors**	**Prediction cases**	**Effect size and 95% interval**			**Test of null (2-trial)**	
		**ES**	**LL**	**UL**	**Z value**	***p***
Traumatic experience	30	1.728	1.496	1.959	14.64	0.000
Problematic parent-child relationship	18	1.585	1.279	1.899	10.16	0.000
Adverse life events	13	2.284	1.324	3.245	4.66	0.000
Sleep disturbance	6	1.734	1.084	2.383	5.23	0.000
Problematic internet use	4	2.023	1.906	2.141	33.84	0.000
Mental health problems	17	1.578	1.187	1.969	7.91	0.000
Negative coping style	6	2.040	1.297	2.783	5.38	0.000
Overall	94	1.734	1.582	1.886	22.35	0.000

*ES, effect size; LL, lower limit; UL, upper limit*.

#### Publication Bias

**Figure 9:**
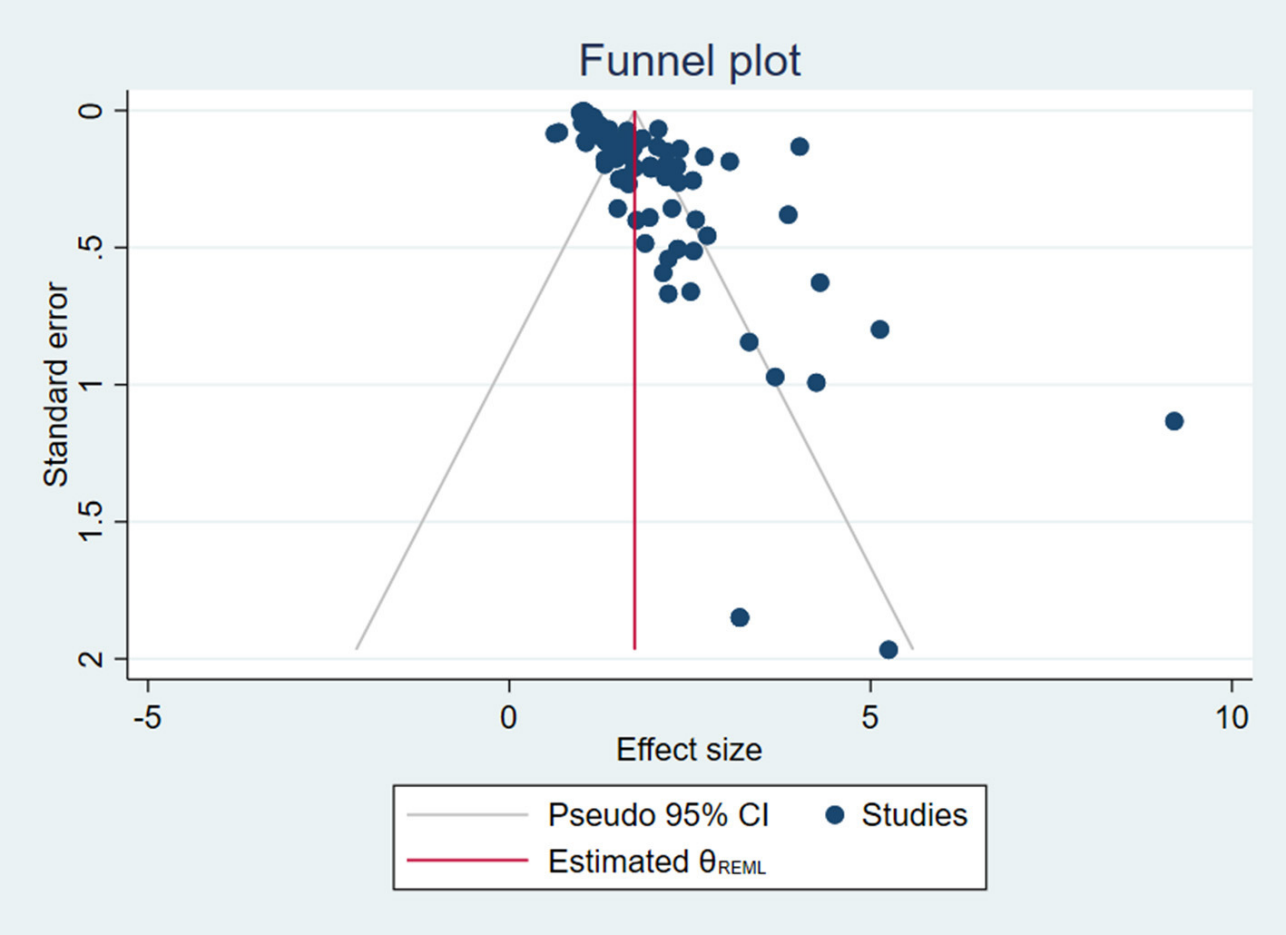
Overall funnel plot.

Egger's test was used to detect publication bias for each factor and NSSI. As shown in Table 3, the factor of internet addiction was the only one without publication bias (*p* = 0.166). However, the Egger's intercepts for traumatic experience, problematic parent-child relationship, adverse life events, lower sleep quality, and low mental health status were all statistically significant, suggesting publication bias (*p* < 0.05). Furthermore, the funnel plot was created to examine publication bias including all factors (see Figure 9). The funnel plot shows that most plots in the graph were distributed around the mean effect size on the top with some discrete plots, showing that the results are acceptable.

**Table 3:** Publication bias test.

**Factors**	**Egger's intercept**	**SE**	***p***
Traumatic experience	3.75	0.634	0.000
Problematic parent-child relationship	2.39	0.605	0.001
Adverse life events	3.41	0.297	0.000
Sleep disturbance	2.10	0.455	0.000
Problematic internet use	−1.29	0.931	0.166
Mental health problems	3.58	1.136	0.002
Negative coping style	5.41	0.852	0.000
Overall	2.97	0.297	0.000

### Moderator Analysis

#### Type of Factors Associated With NSSI

NSSI prediction cases drawn from these seven factors showed significant differences between mental health problems (prediction cases = 17, OR = 1.578, 95% CI: 1.187 to1.969, *p* < 0.001) and negative life events (prediction cases = 13, OR = 2.284, 95% CI: 1.324–3.245, *p* < 0.001). The subgroup analysis was significant [$\chi_{\left(5\right)}^{2}$ = 14.09, *p* = 0.029].

#### Sample Type

Weighted mean odds ratios in the general samples were significantly weaker (prediction cases = 89, OR = 1.672, 95% CI = 1.531–1.813) than left-behind samples (prediction cases = 5, OR = 4.119, 95% CI = 1.578–6.659). The subgroup analysis showed that $\chi_{\left(1\right)}^{2}$ = 3.55, *p* = 0.059. After controlling the effects of factor type, the meta-regression showed that the moderator effect was significant (Coef. = 1.719, SE = 0.457, *p* < 0.001).

## Discussion

NSSI in adolescents has increased in recent years in China (31). However, factors associated with this high-risk behavior are not well documented. To our best knowledge, this was the first attempt to synthesize the factors associated with NSSI among adolescents in China. Understanding the factors associated with NSSI may guide development of screening tools, risk assessments, and targeted therapeutic interventions. Further, determining which variables confer the greatest risk for suicidal behaviors among adolescents with NSSI is critical to identify patients at the highest risk for suicide (32).

Our findings revealed several statistically significant factors associated with NSSI, but the overall effects were weaker than anticipated, and most significant factors did not result in large increases in the absolute odds for NSSI.

Twenty-five full-text articles including 30 separate studies (94 prediction cases) with 186,447 subjects were included in this meta-analysis. The random effect model showed that adolescents with a traumatic experience, problematic parent-child relationship, adverse life events, sleep problems, problematic internet use, mental health problems, and negative coping style had a higher incidence of NSSI.

We examined the magnitude of specific factor categories associated with NSSI. We included the following seven dimensions of associated factors: traumatic experience (adverse childhood experiences, general trauma, physical abuse, emotional abuse, sexual trauma, emotional neglect, indirect aggression, verbal aggression, verbal abuse, and physical abuse), problematic parent-child relationship (neglect, harsh punishment, and intrusiveness), adverse life events (left behind experience, bullying, stressful life event, and academic pressure), sleep disturbance (poor sleep quality and frequent nightmares), problematic internet use (problematic mobile phone or internet use), mental health problems (loneliness, subclinical mental health issues, emotional problems, anxiety, and fear), and negative coping style.

Based on the effect sizes of each factor, the order for the factors were as follows: adverse life events (effect size = 2.284), negative coping style (effect size = 2.040), problematic internet use (effect size = 2.023), sleep problems (effect size = 1.734), traumatic experiences (effect size = 1.728), problematic parent-child relationship (effect size = 1.585), mental health problems (effect size = 1.578). We found that overall risk factor strength was relatively weak. There were significant differences among the seven factors examined.

Our findings are consistent with previous studies regarding mental related factors associated with NSSI among adolescents. Based on 32 longitudinal studies, Plener et al. (33) found that the depressive symptoms and psychological distress were often reported as predictor for NSSI. The meta-analysis conducted by Fox and Franklin showed that prior history of NSSI, cluster B personality, and hopelessness had the strongest effects among specific NSSI-associated factors, but the estimate of the factor in NSSI was weak overall in an absolute sense in prediction of future NSSI, and the estimated degree was higher in adolescent than adults (18). Compared with two published meta-analyses, our research focused on the more detailed mental health related factors associated with NSSI among Chinese adolescents specifically, and provided foundation for future research.

Among the seven factors investigated, adverse life events had the largest predictive value for NSSI in this meta-analysis. Our findings are consistent with previous studies which showed that Chinese left-behind children who faced more stressful life events were more likely to report depression and engage in NSSI (26). A survey of NSSI among Chinese college undergraduates indicated that the chain mediating effects of stressful life events and negative affect play an important role in NSSI (34).

Negative coping styles were the second most important factor associated with NSSI in this meta-analysis. Passive coping styles, such as self-blaming, was found to be associated with NSSI among undergraduate students in China (35). Adolescents who frequently use NSSI exclusively as a coping mechanism for distress are more prone to act on suicidal behaviors (36). A significant association was reported between NSSI and low positive coping style in girls with multiple adverse childhood experiences while NSSI was increased among both girls and boys with a high negative coping style across all adverse childhood experiences (37). Negative coping style may be a target for potential therapeutic interventions to reduce NSSI.

Problematic internet use as a significant factor associated with NSSI among Chinese adolescents is worth special attention. One cross-sectional study on internet addiction and NSSI in China (38) confirmed the association. Both internet addiction and NSSI involve disturbance in impulse control and may be relied upon by adolescents with an avoidant coping style. One survey of 1,634 middle school students in Human province in China indicated that 12% of the adolescents showed signs of Internet addiction, as assessed using the Chinese Internet Addiction Scale (CIAS). Internet addiction is positively correlated with stress and social anxiety and negatively correlated with socio-economic status (39). Internet addiction may allow adolescents to escape from difficult feelings and experiences in their life (40). One study indicated that problematic internet use alone is not a risk factor for NSSI but might become a risk factor in the presence of comorbid psychiatric disorders (41). A systematic review focusing on the relationship between problematic internet use, suicidality and NSSI, and it showed that a higher prevalence of NSSI of the subjects with problematic internet use, with point prevalence varying considerably between 1.6 and 18.7% (42). The link between NSSI and problematic internet use may suggest that negative emotions and cognition combined with inappropriate coping strategies, such as substance abuse and behavioral avoidance, could lead to NSSI (40, 43).

Early traumatic experience is also an important factor associated with NSSI. In a survey of 1,255 Chinese young adults (ages 17 and 26), 83.3% reported childhood physical abuse and 85.9% reported childhood emotional abuse (44). A meta-analysis of childhood maltreatment found that overall childhood maltreatment was associated with NSSI (OR = 3.42, 95% CI = 2.74–4.26), and the effect sizes for maltreatment subtypes ranged from OR 1.84 (95% CI = 1.45–2.34) for childhood emotional neglect to OR 3.03 (95%CI = 2.56–3.54) for childhood emotional abuse (19). Childhood maltreatment and its subtypes were associated with NSSI. Traumatic experiences such as childhood abuse can lead to NSSI by increasing impulsivity, reducing inhibition of negative emotions, and impairing emotional regulation (45).

A problematic parent-child relationship was a factor associated with NSSI. Parental conflicts and parent-child conflicts have been linked to a range of psychological problems in teenagers, such as problematic internet use, aggressive behavior, and poor sleep (46). Additionally, subgroup analysis showed that the effect size of risk factors in left-behind children was larger than that of the general samples.

Poor sleep quality and nightmares were independently associated with NSSI. Research has shown an association between sleep disturbances and suicidal behavior, however, the relation between NSSI and sleep disturbances has rarely been examined (47). Poor sleep and insomnia have been associated with a greater likelihood of recent NSSI and greater NSSI severity (48). Nightmares, but not insomnia, were associated with NSSI while controlling for depressive symptoms, and the relationship between nightmares and NSSI may be mediated by emotional dysregulation (47). Adolescents with higher levels of loneliness were more likely to engage in NSSI, while adolescents with high levels of social support were less likely to engage in NSSI (46).

## Limitations

Several limitations about this study need to be acknowledged. First, although we used strict criteria when selecting eligible studies, since no uniform definition of NSSI is available, the measures of NSSI varied considerably across the 25 included studies, which might affect our findings. Differences in reported prevalence of NSSI may be due to heterogeneous samples, the definition of NSSI, and the assessment tools used. Second, we found significant publication bias in the included studies regarding all associated factors except for internet addiction. This suggests that our findings (the estimated effect sizes) may be inflated, and caution should be exercised when interpreting these results. Third, the retrieval was limited to articles published in English or Chinese languages, although it is likely that there are valuable publications in other languages that we failed to include. Fourth, almost all (except one) included studies were cross-sectional surveys of non-clinical samples. The inherent limitation of cross-sectional studies makes it hard to infer causality of those factors, therefore, we tried to avoid the term risk factor when possible; and when used, it is more often in a statistical sense than a clinical sense. Finally, NSSI in our analysis was treated as a binary variable and all individuals were classified as NSSI group and non-NSSI group. This binary coding, therefore, may lead to misclassification and artificial inflation of the number of people in the NSSI group. Future analysis may need to use continuous NSSI measurement, which may produce significantly stronger NSSI prediction.

## Implications for Clinicians and Future Research

NSSI is an increasing public health problem in adolescents in China. More tools are urgently needed for prevention, screening, assessment, and treatment of NSSI (49). Many of the identified associated factors for NSSI are modifiable, such as traumatic experiences, mental health symptoms, and sleep disturbance. Further, left-behind children appear uniquely vulnerable and may be disproportionately impacted by certain risk factors. Most of the current domestic studies are cross-sectional surveys, which cannot infer the causal relationship between the associated factors and NSSI. Long-term cohort studies may better elucidate the relationships between specific risk factors, NSSI, and suicide. Using continuous measures of NSSI in future research may be better suited for identifying meaningful risk factors for these behaviors (18). Future studies need to investigate specific vulnerable subgroups, evaluate longitudinal cohorts, and assess the efficacy of interventions to identify, prevent, and treat NSSI.

## Conclusion

Despite some limitations, this study is the first attempt to examine the factors associated with NSSI among Chinese adolescents. The high incidence of NSSI among Chinese adolescents is concerning. A better understanding of this behavior and its associated factors may inform strategies to reduce the incidence of NSSI. Based on a meta-analysis of published reports, we highlighted a few important factors that are associated with NSSI. Further research and interventions addressing problematic parent-child relationships, problematic internet use, and sleep may be important to improving the mental health of adolescents. Only children and left-behind children in China must be further studied. Efforts to de-stigmatize and increase access to mental health treatment are critical. Schools may be engaged in teaching mindfulness and other skills that promote positive coping and emotional regulation. Encouraging children and families to seek mental health treatment for adolescents with NSSI may ultimately decrease the risk of serious long-term consequences, such as suicide.

## Data Availability Statement

The original contributions presented in the study are included in the article/Supplementary Material, further inquiries can be directed to the corresponding author/s.

## Author Contributions

JL and Y-yF designed the study and wrote the protocol. Y-yF and Y-yZ conducted literature searches. Y-yF conducted meta-analysis and produced figures. Y-yF, JL, and Y-yZ wrote the first draft of the manuscript. Y-lT and RC contributed to data interpretation and critical revision of the manuscript. All authors contributed to the article and approved the submitted version.

## Funding

This study was funded by Capital clinical characteristic application research and achievement promotion: clinical study of transdiagnostic group cognitive behavioral therapy for emotional disorders (Grant Number: Z161100000516066) and Beijing Municipal Administration of Hospitals Clinical medicine Development of special funding (Grant Number: ZYLX201815).

## Conflict of Interest

The authors declare that the research was conducted in the absence of any commercial or financial relationships that could be construed as a potential conflict of interest.

## Publisher's Note

All claims expressed in this article are solely those of the authors and do not necessarily represent those of their affiliated organizations, or those of the publisher, the editors and the reviewers. Any product that may be evaluated in this article, or claim that may be made by its manufacturer, is not guaranteed or endorsed by the publisher.
