# Supplementary material for: Factors Associated With Non-suicidal Self-Injury in Chinese Adolescents: A Meta-Analysis
Source: Front Psychiatry. 2021 Nov 30;12:747031. doi: 10.3389/fpsyt.2021.747031 (PMC8669619; doi:10.3389/fpsyt.2021.747031)
Supplement: Supplementary file 1 [file Table_1.DOCX]

Supplemental Table 1. Characteristics in studies evaluating the risk of NSSI

| **No.** | **Authors** | **Publication years** | **Study Design** | **City/**  **Province** | **Period** | **Sample Size**  **N** | **Number of NSSI**  **n(%)** | **Sample Age**  **M(SD)** | **% of girls**  **n(%)** | **Study covariates in multivariate analysis** |
| --- | --- | --- | --- | --- | --- | --- | --- | --- | --- | --- |
| 1 | Xu | 2011 | randomly selected | Anhui | 12months | 3402 | 1027（30.2%） | 14. 02(1.21) | 1657(48.7%) | Left-behind, relationship with father, achievement, source of psychological control, somatization, hostility, terror |
| 2 | Xu | 2012 | cluster sampling | China | 12months | 12113 | 2378（19.6%） | 15.59(2.77) | 6334(52.3%) | Psychological sub-health state, emotional problems, conduct problems, social adjustment difficulties |
| 3 | Yan | 2012 | multistage sampling | Sichuan | 12months | 1288 | 292(22.67%) | 14.24(4.27) | 583(45.26%) | Social life-events, depression, social support, family cohesion, family conflict, family harmony |
| 4 | Tang | 2013 | randomly selected | Guangdong | 12months | 2907 | 345(11.9%) | 15.4 (1.8) | 1471(50.6%) | Verbal aggression, hostility, indirect aggression, emotional management, one child, gender, satisfaction of appearance |
| 5 | Zhang | 2014 | cluster sampling | Shanghai | 12months | 2400 | 521(21.70%) | 13.38(0.95) | 1180(49.1%) | Sex, Common trauma, physical abuse experience, emotional abuse, sexual trauma experience, poor parent-child relationship, non-parental primary parenting before age 6, anxiety symptoms, social support (protective factors) |
| 6 | Su | 2015 | Stratified cluster sampling | Guiyang | 12months | 4617 | 2046(44.3%) | 15.20(1.81) | 2499(54.1%) | Experience of emotional abuse, physical abuse, sexual abuse, emotional neglect, physical neglect, and abuse |
| 7 | Zhang | 2016 | convenient cluster sampling | China | 12months | 25378 | 6971(27.5%) | 15.18(1.79) | 13053(51.4%) | Psychological symptoms, health literacy |
| 8 | Zhang | 2016 | random cluster sampling | Linyi | 6 months | 3957 | 1092(27.6) | 15.8 (1.9) | 2116(53.5%) | Gender, Family residence, only child or not, school performance, metal health, stressful life events, severe punishment from father, excessive intervention from father, excessive intervention and protection from mother, excessive intervention from mother |
| 9 | Zeng | 2016 | cluster sampling | Wuhan | 12months | 1808 | 330(18.3%) | 14.2(0.8) | 831(46.0%) | parental neglect, estrangement between parents and children, estrangement between parents |
| 10 | Gu | 2016 | Stratified cluster random sampling | Bengbu | 12months | 5149 | 1670(32.4%) | 15.06 (1.36) | 2295(44.6%) | Sex, School segment, household registration, the only child or not, mental health status, sleep belief |
| 11 | Liu | 2017 | cross-sectional | Shandong | 12months | 2090 | 184(8.80%) | 15.5 (2.1) | 1063(50.9 %) | Age, impulsiveness scale score, externalizing problem scale score, sleep quality, nightmares last year |
| 12 | Xin | 2017 | cross-sectional | China | 12months | 11880 | 3563(30%) | 14.62(1.90) | 5999(50.5 %) | History of smoking, truancy, binge drinking, running away from home, Fighting, unsafe sex, physical inactivity, suicide ideation, suicide plan, suicide attempt, impulsivity, depression, anxiety |
| 13 | Fu | 2018a | convenience sampling | Jiangxi | 12months | 3831 | 1293（33.75%） | 15.48(1.65) | 1612（42.08%） | Gender, domicile, scolding of elders, corporal punishment, study burden, venting emotions, fantasy (denial), emotional symptoms, moral problems |
| 14 | Fu | 2018b | convenience sampling | Jiangxi | 12months | 3298 | 1110（33.66%） |  | 1367（41.45%） | Gender, scolding of elders, corporal punishment of elders, academic pressure, patience, venting emotions, fantasy (denial), emotional symptoms, hyperactivity attention disorder |
| 15 | Jiang | 2018 | cluster sampling | Jiangxi | 12months | 1810 | 123(6.80%) | N/A | 805 (44.48%) | Age, sex, Smoking, drinking, bullying at school, sexual orientation, stress at school |
| 16 | Ma | 2018 | Stratified cluster sampling | Mainland | 12months | 9704 | 3740(38.5%) | N/A | 5105(52.6%) | Sex, Grade, geographical region, family financial status, learning burden, childhood abuse, psychopathological symptoms, positive coping style, negative coping style, impulse control |
| 17 | Ru | 2018a | convenience sampling | Jiangxi | 12months | 3831 | 1293(33.8%) | 15.48(1.65) | 2929(41.8%) | Attachment: The relationship between parents (trust, communication, alienation) |
| 18 | Ru | 2018b | convenience sampling | Jiangxi | 12months | 3298 | 1110(33.7%) |  |  | Attachment: The relationship between parents (trust, communication, alienation) |
| 19 | Li | 2019 | cross-sectional | China | 12months | 22628 | 7264(32.10%) | 15.36(1.79) | 11638(51.4%) | Problematic mobile phone use, health literacy |
| 20 | Liu | 2019 | longitudinal | Shandong | 12months | 7072 | 621(8.8%) | 14.59 (1.45) | 3536(50.0%) | Nocturnal sleep time, insomnia symptoms, sleep quality, nightmares in the past year |
| 21 | Wan | 2019a | cross-sectional | China | 12months | 7440 | 3868(26.10%) | 15.46(1.8) | 7440(100%) | Adverse childhood experiences, social support |
| 22 | Wan | 2019b | cross-sectional | China | 12months | 7380 |  | 15.41(1.8) | 0(0%) | Adverse childhood experiences, social support |
| 23 | Xiao | 2019 | cross-sectional | Yunnan | 12months | 2619 | 1269 (48.5%) | 14.01 (1.79) | 1277 (48.76%) | Sex, education level, parents’ education level, depression, resilience |
| 24 | Cao | 2019 | cross-sectional | Shenzhen | 12months | 2104 | 230(11.6%) | N/A | 1029(48.9%) | Experience of school bullying, depression, Internet addiction |
| 25 | Ma | 2019 | random cluster sampling | China | 12months | 15538 | 4459(28.70%) | 15.13(1.82) | 7539(48.5%) | Gender, age, education level, ethnicity, parents' education level, family type, parenting style, only child, family history of mental illness, loneliness, Internet addiction |
| 26 | Xia | 2019a | cross-sectional | Beijing | 12months | 650 | disable:123(18.9%) | 12.0(n/a) | 488(37.5%) | Disability, gender, ethnicity, age, parents’ education level, sleep duration, difficulty falling asleep, after midnight |
| 27 | Xia | 2019b | cross-sectional | Beijing | 12months | 650 | nornal:56(8.6%) |  |  |  |
| 28 | Wan | 2020 | cross-sectional | Zhengzhou, Guiyang | 12months | 9704 | 3740(38.54%) | 15.59(1.80) | 5104(52.60 %) | Gender, age, school, parents’ education level, economic status of family, psychological symptoms, adverse childhood experience, positive coping style, negative coping style. |
| 29 | Pang | 2020 | cross-sectional | Guangxi | 12months | 14822 | 4527(30.54%) | 15.27 (1.94) | 7174(48.40%) | Education level, the only child or not, family income, family type, educational mode, family history of mental illness, internet addiction |
| 30 | Wang | 2020 | stratified cluster sampling | China | 12months | 14500 | 1379(9.5%) | 14.83(1.79) | 7153(49.33%) | Childhood abuse (sexual abuse, emotional abuse, and physical abuse.) |

Supplemental Table 2. Quality assessment of included cross-sectional studies (Agency for Healthcare Research and Quality)

| Study | Publication year | 1) Define the source of information (survey, record, review) | 2) List inclusion and exclusion criteria for exposed and unexposed subjects (cases and controls) or refer to previous publications | 3) Indicate time period used for identifying patients | 4) Indicate whether or not subjects were consecutive if not population-based | 5) Indicate if evaluators of subjective components of study were masked to other aspects of the status of the participants | 6) Describe any assessments undertaken for quality assurance purposes (e.g., test/retest of primary outcome measurements) | 7) Explain any patient exclusions from analysis | 8) Describe how confounding was assessed and/or controlled. | 9) If applicable, explain how missing data were handled in the analysis | 10) Summarize patient response rates and completeness of data collection | 11) Clarify what follow-up, if any, was expected and the percentage of patients for which incomplete data or follow-up was obtained | Quality score |
| --- | --- | --- | --- | --- | --- | --- | --- | --- | --- | --- | --- | --- | --- |
| Xu | 2011 | 1 | 1 | 0 | 0 | 1 | 0 | 0 | 1 | 1 | 1 | 0 | 6 |
| Xu | 2012 | 1 | 1 | 1 | 1 | 1 | 0 | 0 | 1 | 1 | 1 | 1 | 9 |
| Yan | 2012 | 1 | 1 | 1 | 0 | 1 | 0 | 0 | 1 | 1 | 1 | 0 | 7 |
| Tang | 2013 | 1 | 1 | 1 | 0 | 1 | 0 | 0 | 1 | 1 | 0 | 0 | 6 |
| Zhang | 2014 | 1 | 1 | 0 | 0 | 1 | 0 | 0 | 1 | 1 | 0 | 0 | 5 |
| Su | 2015 | 1 | 1 | 0 | 0 | 1 | 0 | 0 | 1 | 1 | 0 | 0 | 5 |
| Zhang | 2016 | 1 | 1 | 1 | 0 | 1 | 0 | 0 | 1 | 1 | 1 | 0 | 7 |
| Zeng | 2016 | 1 | 1 | 0 | 0 | 1 | 0 | 0 | 1 | 1 | 1 | 0 | 6 |
| Gu | 2016 | 1 | 1 | 1 | 0 | 1 | 0 | 0 | 1 | 1 | 1 | 0 | 7 |
| Liu | 2017 | 1 | 1 | 1 | 0 | 1 | 0 | 0 | 1 | 1 | 1 | 0 | 7 |
| Xin | 2017 | 1 | 1 | 0 | 0 | 1 | 0 | 0 | 1 | 1 | 0 | 0 | 5 |
| Fu | 2018 | 1 | 1 | 1 | 0 | 1 | 0 | 0 | 1 | 1 | 0 | 0 | 6 |
| Jiang | 2018 | 1 | 1 | 1 | 0 | 1 | 0 | 0 | 1 | 1 | 1 | 0 | 7 |
| Ma | 2018 | 1 | 1 | 0 | 0 | 1 | 0 | 0 | 1 | 1 | 1 | 0 | 6 |
| Ru | 2018 | 1 | 1 | 0 | 0 | 1 | 0 | 0 | 1 | 1 | 1 | 0 | 6 |
| Li | 2019 | 1 | 1 | 1 | 0 | 1 | 0 | 0 | 1 | 1 | 0 | 0 | 6 |
| Wan | 2019 | 1 | 1 | 1 | 0 | 1 | 0 | 0 | 1 | 1 | 1 | 0 | 7 |
| Xiao | 2019 | 1 | 1 | 1 | 0 | 1 | 0 | 0 | 1 | 1 | 1 | 0 | 7 |
| Cao | 2019 | 1 | 1 | 1 | 0 | 1 | 0 | 0 | 0 | 1 | 1 | 0 | 6 |
| Ma | 2019 | 1 | 1 | 0 | 0 | 1 | 0 | 0 | 1 | 1 | 0 | 0 | 5 |
| Xia | 2019 | 1 | 1 | 1 | 0 | 1 | 1 | 0 | 1 | 1 | 1 | 0 | 8 |
| Wan | 2020 | 1 | 1 | 1 | 0 | 1 | 0 | 0 | 1 | 1 | 1 | 0 | 7 |
| Pang | 2020 | 1 | 1 | 1 | 1 | 1 | 0 | 0 | 0 | 0 | 1 | 0 | 6 |
| Wang | 2020 | 1 | 1 | 1 | 1 | 0 | 1 | 0 | 1 | 0 | 1 | 0 | 7 |

Notes: 1 means the study meets this criterion, 0means the study does not meet this criterion. Quality score no less than 5 will be included in this meta-analysis.

Supplemental Table 3. Quality assessment of included longitudinal studies (Newcastle Ottawa Scale)

|  | Selection | | | | Comparability | Outcome | | |  |
| --- | --- | --- | --- | --- | --- | --- | --- | --- | --- |
| Study | Representativeness of exposed cohort | Selection of non-exposed cohort | Ascertainment of exposure | Demonstration that outcome of interest was not present at start of study | Comparability of cohorts on the basis of the design or analysis | Assessment of outcome | Was follow-up long enough for outcomes to occur (mean follow- up) | Adequacy of follow up of cohorts | Quality score |
| Liu,2019 | 1 | 0 | 0 | 1 | 1 | 1 | ★（1 years） | 0 | 5 |

Notes: 1 means the study meets this criterion, 0 means the study does not meet this criterion. Quality score no less than 5 will be included in this meta-analysis.
